# What Is New in Listeriosis?

**DOI:** 10.1155/2014/358051

**Published:** 2014-04-14

**Authors:** Almudena Hernandez-Milian, Antoni Payeras-Cifre

**Affiliations:** Hospital Son Llàtzer, Carretera de Manacor Km4 07198, Majorca, Spain

## Abstract

Listeriosis is a disease caused by *Listeria monocytogenes (L. monocytogenes). L. monocytogenes* is bacteria that usually infects some determined inhabitants, especially high risk patients such as the elderly, immunosuppressed patients and pregnant women. However, it can also affect people who do not have these risk factors. *L. monocytogenes* is widespread in nature being part of the faecal flora of many mammals and *it is a common foodborne source.* It is acquired by humans primarily through consumption of contaminated food. Besides, between 1% and 10% of the population is a faecal carrier of *L.monocytogenes*. Listeriosis may occur sporadically or in outbreaks. Infection causes a spectrum of illness, ranging from febrile gastroenteritis to invasive disease, including bacteraemia, sepsis, and meningoencephalitis. This infection has a low incidence, although it is undeniably increasing, particularly due to the rise of population of over 60 years old or of under 60 years olds with a predisposing condition. The diagnosis is complicated because of its incubation period and the different clinical manifestations. Also listeriosis has a high mortality despite adequate and early treatment. The importance of bacteraemia for *L. monocytogenes* lies in the infrequency of this bacterium and the high mortality, even with appropriate antibiotic treatment.

## 1. Microbiology

*Listeria monocytogenes* is a gram-positive bacillus and facultative intracellular bacterium [1].* L. monocytogenes* was first described in 1926 by Murray et al. while they were investigating an epidemic infection among laboratory rabbits and guinea pigs [2]. Despite the considerable confusion in the nomenclature of the pathogen, the official name of* Listeria monocytogenes* was adopted in 1940 [2] by the Sixth Edition of Bergey's Manual of Determinative Bacteriology [3].

The first case of human listeriosis was reported in 1929 by Nyfeldt [4]. Several years later, during the 1980s, the increased number of reported cases of listeriosis in several countries turned into a recognized disease of foodborne [5].

## 2. Epidemiology

*L. monocytogenes *has been considered a widespread bacterium in nature, as it is part of the faecal flora of many mammals and it is a common foodborne source. It is believed that the main route of bacterial transmission occurs through the consumption of contaminated food such as meat (sausages, pate, ham, salami, and chicken), vegetables, ready-to-eat seafood (such as smoked fish or mussels), raw seafood, unpasteurized milk, soft-serve ice creams, and soft cheeses. Also, due to the ability to survive and grow in harsh conditions such as wide pH range, high salt concentration, and temperatures between −2 and –42 degrees C, it makes of this pathogen a high concern to the food industry [6–9].

The incubation period of listeriosis is variable. The first published records about the incubation periods of* L. monocytogenes* were documented in the New England Journal of Medicine during a large listeriosis outbreak in 1988 by Linnan et al. (range: 3 to 70 days) [10]. It is related to multiple aspects. Firstly,* L. monocytogenes* can contaminate a large variety of foods. Secondly, the incubation period is variable and long, ranging from 1 to 70 days [8]; in pregnancy-associated cases, the authors reported a longer incubation period (median 27.5 days, ranging from 17 to 67 days) than central nervous system cases (median 9 days, ranging from 1 to 14 days) and bacteraemia cases (median 2 days, ranging 1 to 12 days). Also, in febrile gastrointestinal disease, the median of the incubation period was reported to be 24 hours, ranging from 6 hours to 10 days. Thirdly, many products can retain the bacteria during several days or weeks and, therefore, can be eaten by the patient on multiple occasions [7, 8, 10].

## 3. Pathogenesis

*L. monocytogenes* uses various host proteins, including some internalins to adhere and to invade the host cells. In the infected hosts,* L. monocytogenes* has the ability to induce its own entry into host cells. Once it is in the intracellular phagocytic vacuole, the bacteria secrete listeriolysins and phospholipases that allow it to lyse the vacuolar membrane and avoid the intracellular killing. Subsequently, adjacent cells have been invaded through plasma membrane protrusions and therefore cell-to-cell spread occurs. Through this cycle,* L. monocytogenes* can move from one host cell to another, without being in the extracellular environment, thus escaping to the human T-cell immune system [11]. The invaded cells can cross the intestinal epithelium barrier and also other tissues and organs such as the liver. The surviving bacteria are replicated in hepatocytes, and early recruitment of polymorphonuclear cells leads to hepatocyte lysis and thereby the bacterial release. The majority of bacteria can become trapped in the liver and after that some bacteria can rapidly enter the blood system and can invade the mesenteric lymph nodes. If the infection is not controlled at the stage in which the bacterium is in the liver, for instance, due to a severe immunodepression, a secondary bacteraemia can be developed. [11]. Bacteria are circulating in the blood, either free or associated with leucocytes, and are disseminated to the preferred sites of the* L. monocytogenes* with transgressing the blood-brain barrier or the placental barrier [12]; this predisposition is a consequence of suppressed T-cell mediated immunity. This is the cause of the severity of listeriosis despite the proper treatment. Similarly, pregnant women, the elderly, and those receiving immunosuppressive therapy are also at risk because of the impaired or modulated immune function [7].

The main host defense against listeriosis is the cell mediated immunity, and therefore individuals with T-cell dysfunction seem to be particularly at risk of contracting the disease. In addition, pathogen-specific factors also seem to be important in causing the disease [13]. The disease has been well-defined in particular inhabitants, such as the elderly, the immunocompromised patients (e.g., those who are receiving corticosteroids or chemotherapy), patients in hemodialysis, those with transplants and diabetes, HIV carriers, drug dependents, pregnant women, and newborn infants; in those cases, an increased risk of invasive listeriosis exists. This is characterized by bacteraemia, meningitis, foetal loss, and death [13]. However, it can also affect people who do not have any of these risk factors [14]. Therefore, it is also important to note that these patients are particularly at risk of developing symptomatic disease [15, 16] although the invasive illness can also occur in persons with competent immune systems [17].

A study in England and Wales showed that the population that suffered a bloodstream infection of* L. monocytogenes* has changed during 2001–2008; more patients over 60 years old had more episodes of bacteraemia than the reported in previous years. A total of 1.413 patients were included in the study; malignancies (especially of the blood), kidney and liver disease, diabetes, alcoholism, and age over 60 years old were associated with an increased risk for listeriosis [18].

*L. monocytogenes* can also manifest as outbreak. Although most cases are sporadic, the investigation and analysis of the foods that can transmit the infection and that can provide insights into the pathogens, food vehicles, and food-handling practices associated with foodborne infections could decrease the number of outbreaks [19].

*L. monocytogenes* has been differentiated into 13 serotypes; serotypes 1/2a, 1/2b, and 4b have been involved in the majority of reported human listeriosis cases (more than 95%) [7]. Most isolates from food belong to serogroup 1/2 and predominate in cases of sporadic listeriosis, but most of the outbreaks of human disease are caused by strains of serotype 4b. It has been suggested that many outbreaks have been caused by a small number of* L. monocytogenes* epidemic clones, that is, by a closely related group of isolates that evolved clonally [7].

There is a report which summarizes listeriosis outbreaks reported to the Foodborne Disease Outbreak Surveillance System of the Centers for Disease Control and Prevention during 1998–2008. Twenty-four confirmed listeriosis outbreaks were reported in those years resulting in 359 illnesses, 215 hospitalizations, and 38 deaths. Serotype 4b caused the largest number of outbreaks cases [20, 21]. Another well-known outbreak of* L. monocytogenes* was associated with cantaloupe, Colorado (USA), where more than 30 people died. The environmental samples that were collected during the investigation yielded insolate matching outbreak-related subtypes, confirming that the whole cantaloupe produced by a single Colorado farm was the outbreak source. Unsanitary conditions identified probably were the cause in the contamination of the cantaloupes with* L. monocytogenes* [22]. After this outbreak, a survey in the USA investigated a nationwide listeriosis outbreak that occurred from August to October of 2011; they identified 147 outbreak-related cases in 28 states. The majority of the patients (127 out of 147, 86%) were 60 years of age or older. Thirty-three of the 147 patients (22%) died. Patients, who have eaten cantaloupe, were significantly more likely to have the outbreak-related illness [23].

Few cases have been reported between 1999 and 2011 of healthcare-associated transmission of* L. monocytogenes* via contaminated foods, healthcare workers, and infected patients; however, most of these cases were clustered in time and space [6].

It should be noted that, since more than 20 years, investigators have recognized that* L. monocytogenes* can be carried in the gastrointestinal tract. It can be isolated in 1%–10% of the stool of the population, where it can persist without causing any symptoms. Also,* L. monocytogenes* can be detected in the faeces of nearly 70% of healthy nonpregnant women and 44% of pregnant women [6, 24].

## 4. Clinical Manifestations

The infection by* L. monocytogenes* in healthy individuals usually causes self-limited febrile diarrhea or can be asymptomatic; however, in immunosuppressed individuals, it can cause clinical episodes of invasive listeriosis [25].

The disease has three major invasive clinical presentations: bloodstream infection, infection of the central nervous system, and maternal foetal listeriosis. In adults, the most common clinical form of listeriosis is meningitis, due to the bacterial tropism to the central nervous system [26]. It has been suggested that individuals with predisposing factors seem to suffer more episodes of septicemia than of meningitis. But this situation may be due to the strict follow up of these individuals by the health system requesting more frequently sterile cultures [19].

The most frequent manifestation in immunocompromised individuals is the bacteraemia with no obvious focus; and therefore it is very difficult to suspect and consequently to diagnose a bacteraemia by* L. monocytogenes*. Clinical manifestations of bacteraemia in this kind of patients are not easy to diagnose, because usually the host factors do not permit expressing the illness in immunocompromised patients as healthy inhabitants. It is very important to make a good history and complete exploration. It is also recommended when the medical record to the patient is performed to classify the severity of the illness in accordance with the inflammatory response syndrome definition and sepsis criteria above all, if the patient presents risk factors for invasive infection by* L*.* monocytogenes* [27].

Likewise, clinical symptoms of* L. monocytogenes* bloodstream infections are similar to other etiologic agents causing bacteremia, such as fever, myalgia, and general malaise. They are not uncommon signs and symptoms such as headaches, abdominal pain, vomiting, and diarrhea, which indicate that the microorganism has been inside of the digestive tract before getting into the bloodstream. Finally the infection can progress to a septic shock [19, 28, 29].

The mortality of this pathological entity is high and variable, ranging from 20% to 30% depending on the authors [25]. There were some studies that reported the mortality of listeriosis in different countries.

In the United States (USA),* L. monocytogenes* is rare. However, it is the third leading cause of death by foodborne. From 2009 to 2011 in the USA, a total of 1,651 invasive listeriosis cases were reported, of which 1,308 (79%) were bloodstream infections and 292 (17.6%) inhabitants died [30].

In a study from China (1964-2010) they found a total of 147 clinical cases of* L*.* monocytogenes* and 68 (46%) were septicaemia. The overall case-fatality rate was 26% [31].

Most of the patients in a study from Denmark (1994–2003) with bloodstream infection were predisposed to suffer the disease because of concurrent underlying illness. Besides, half of the patients were aged over 70 years and 21% of them died due to the disease [13].

Another study in England and Wales has calculated the risk for nonpregnancy-associated listeriosis cases reported to the national surveillance system in England during 1999–2009. A total of 479 patients died [18].

In our own experience, we have published a study about the infections by* L. monocytogenes* in the Balearic Islands (Spain) that were detected between 2002 and 2013. Eighteen (50%) patients that suffered from primary bacteraemia and positive blood culture were detected in 31 cases. Overall mortality was 50% (18 cases) and in 9 cases (25%) was related to* L. monocytogenes* [32].

Primary bacteraemia was also the most common clinical presentation of* L. monocytogenes* in a study from Madrid (Spain, 1986–2007) and also increased until 55.7% in the last years of the study. Mortality was 24.3%, similar to other series [33].

In another study from Spain (Barcelona, 1991–2005), primary bacteraemia was found in 63 cases from 110 episodes and it was more frequent in inhabitants with haematological disease. The reported mortality rate was 14%, most of them for CNS disease [34].

The variability of mortality can be explained by the risk factors of the host and medical history of the patient. The incidence of mortality in different countries can be observed in Table 1. But also, another explanation could be that sometimes the patients present to an insidious clinic with also symptoms compatible with other diseases and as a result of this a delay in the diagnosis of listeriosis occurs [35].

However, because of its high case-fatality rate, listeriosis is, after salmonellosis, the second most frequent cause of foodborne infection-related deaths in Europe and USA [19].

## 5. Incidence of *L. monocytogenes*

It has been reported that the annual incidence of listeriosis ranges between 0.1 and 1 case per 100.000 inhabitants. Although listeriosis is less common than other foodborne diseases, 19% and 17% of the known causes of foodborne disease-related deaths occurring in the USA and France, respectively, are caused by* L. monocytogenes*. Indeed, listeriosis was not a notifiable disease in the USA until 2000 and its diagnosis might be underestimated [36]. In recent years, the annual number of reported listeriosis cases has been increased in several European countries. Goulet et al. suggested that this increase might be due to the increase of population of over 60 years old or of under 60 years old with an immunocompromised predisposing condition [12].

In a report from 20 countries (1991), 782 cases of listeriosis were reported. It showed that 43% of the infections were related to pregnancy, 29% to septicemia, and 24% to central nervous system infection and 4% were atypical forms of the infection [37].

In the USA, the incidence of listeriosis decreased by 24% from 1996 through 2001, but since then there has been an increased incidence [36]. Silk et al. have reported an incidence rate of listeriosis of 0.27 cases per 100,000 US inhabitants between 2004 and 2009 [14]. From 2009 to 2011, the average annual incidence of listeriosis in the USA was 0.29 cases per 100,000 inhabitants and in adults aged over 65 years the incidence increased 1.3 cases per 100,000 inhabitants [30].

In Taiwan during 1996–2008, a total of 48 patients with listeriosis were identified. Average annual incidence increased from 0.029 to 0.118 cases per 1,000 admissions before and after January 2005 [38].

Listeriosis is not yet a notifiable disease in China, and there is no national monitoring system for those cases. In a study in China from 1964 to 2010, they were reported 479 isolated of* L*.* monocytogenes*, and 82 of them were outbreak-related cases [31].

**Figure 1:**
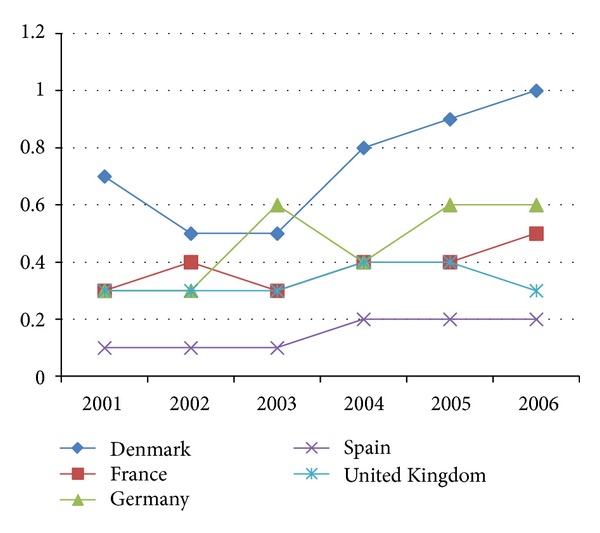
Incidence of listeriosis per 100.000 inhabitants in five countries of the European Union from 2001 to 2006.

In Europe, there are also some studies. Denny and McLauchlin conducted a study comparing the incidence by* L. monocytogenes* in Europe. The impact of the incidence of listeriosis in different countries between 2001 and 2006 can be observed in Figure 1 [39].

A review paper about invasive listeriosis in Denmark from 1994 to 2003 showed a total of 299 invasive cases of listeriosis. Two-thirds of the cases were caused by isolates of serogroup 1/2, while the other one-third was caused by serogroup 4. The incidence of listeriosis in Denmark peaked in 2003 at 0.46 cases/100 000 inhabitants [13].

In 2011, 28 EU/EEA countries provided data on the disease and reported an average case rate of 0.31 per 100,000 population [40]. The highest rates were observed in Denmark (0.88 per 100,000). Most of the patients were over 65 years old (867 (57%)). Also they noticed a seasonal trend for listeriosis in 2011; the first peak was in May, the second was in August, and the third one was in November [40].

Hernandez-Milian et al. have conducted a study in Spain (Balearic Island) to examine the incidence of* L. monocytogenes* in our area from 2002 to 2013. We have included 44 microbiological isolates of* L. monocytogenes *in 36 patients; 41.6% of the patients were over 65 years old. Outbreaks were not observed. In 5 cases, the infection was hospital-acquired and most of the total of the patients had a hematologic disease. It is important to note that in Balearic Island it is not a mandatory declaration disease [32].

In one study from Madrid (Spain), they detected 111 cases of listeriosis between 1986 and 2007. Incidence of listeriosis gradually increased over the years (from 0.46/100.000 inhabitants to 1.03/100.000 inhabitants; *P* = 0.001) [33].

In Barcelona (Spain), 110 cases for bacteraemia of* L. monocytogenes* from 1991 to 2005 have been reported. Most of the patients affected were men, 7 were pregnant women, and 2 of them suffered fetal deaths. Almost 90% of the cases presented with an immunosuppressed associated disease [34].

In another study from Valencia (Spain), 58 cases of* L. monocytogenes* bloodstream infection were reported from 2008 to 2010 [41].

The increased incidence of the disease could be related to higher incidence of concomitant diseases, with treatments more associated to immunosuppression, and all at the same time in relation to the increased survival of the ill population. Perhaps, in lesser measure, the increase of the incidence is due to an elderly population [14, 42].

Pouillot et al. demonstrated in a recent study in the USA between 2004 and 2009 that a significant increase in listeriosis incidence begins among persons as young as 45 years and subsequently the incidence rate increases steadily with age [43].

In Europe, invasive listeriosis has been reported to be an infection of great concern to public health due to its clinical severity (hospitalization rate > 90%) and high fatality rate (20% to 30%), despite the correct treatment and its low incidence (0.4 cases per 100 000 population) [27].

A comparison of the incidence of* L. monocytogenes* in different countries discussed in earlier studies can be observed in Table 2.

**Figure 2:**
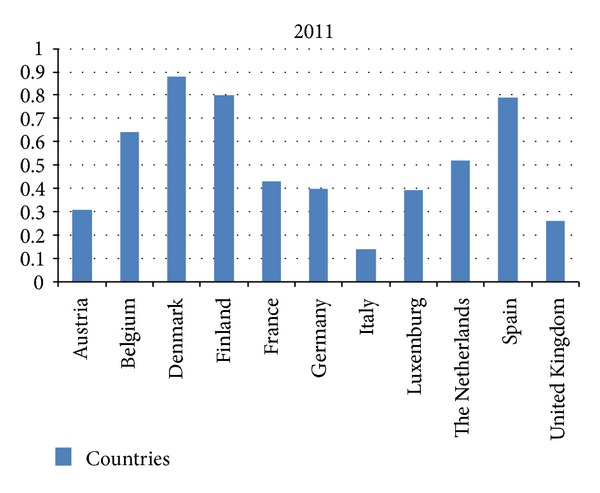
Summary report of cases of* L. monocytogenes* per 100.000 inhabitants in the European Union in 2011.

The European Centre for Disease Prevention and Control and the European Food Safety Authority have made a summary report on the incidence of* L. monocytogenes* with the confirmed cases in the European Union in 2011 (see Figure 2) [40].

## 6. Diagnosis

Sometimes, making a diagnosis of listeriosis is difficult. The most important point to make a diagnosis of* L. monocytogenes* will be to suspect the presence of the bacteria and take it into account. As we have previously discussed, it is necessary to make a good medical record considering the host factors and the clinical symptoms. After that it is important to collect sterile samples where* L. monocytogenes* grows in particularly blood cultures [19]. The bacteraemia can be classified as primary or secondary; therefore it would be also important to collect other samples as joint, peritoneal, and cerebrospinal fluid according to clinical manifestations. In addition to the host factors, the pathogen-specific factors also seem to be important in causing disease. It has been suggested that bacteraemia is more likely to be diagnosed in patients with underlying conditions, because these patient groups are mostly monitored with blood cultures, whereas mild cases of bacteraemia (e.g., influenza-like disease) or in healthy individuals are likely to remain undetected. Another factor to consider is that a neurological or abdominal affectation by* L. monocytogenes *might end up in bacteraemia with all related complications [19].

The identification of* L. monocytogenes* is performed using standard microbiological techniques. The bacteria grow in 24–48 hours, forming small rounded colonies and they present *β*-hemolytic reaction on blood agar [19]. Because it shares some characteristics with other species of gram-positive bacteria, one should establish the differential diagnosis with some of them as* Streptococcus* [36]. It should be noted that the anti-listeriolysin O antibodies have not been proved useful in the diagnosis of acute invasive infection [19].

## 7. Treatment

Listeriosis often requires antimicrobial therapy; however, in the cases of bacteraemia, it is important to start treatment as early as possible, because of the severity of the disease and the high associated mortality.

The choice of treatment consists of a *β*-lactam antibiotic, normally ampicillin. Because the penicillins are bacteriostatic, some studies have attempted drug combinations. For example, the simultaneous use of ampicillin and an aminoglycoside (usually gentamicin) is one of the most useful methods, especially in patients over age 50 [36, 44]. The dose is important in the treatment of invasive disease, which requires a dose of 6 grams or higher [44].

Recommended second-line agents in case of allergy to *β*-lactams or in certain disease states are trimethoprim/sulfamethoxazole, erythromycin, vancomycin, and fluoroquinolones. A low resistance of isolates of* L. monocytogenes* to these antibiotics was found [36].

The duration of treatment of bacteraemia is usually about two weeks, in accordance with the clinical evolution. But the appropriate duration of treatment is not clear. After two weeks of treatment, there have been reported recurrences in immunocompromised patients. Therefore, it seems appropriate to prolong the time of therapy in such cases depending on the clinical manifestations of the patients [36].

## 8. Prevention

In 2011, contaminated cantaloupes from a single farm caused the deadliest US foodborne disease outbreak in the last 90 years as we have before discussed. Public health officials rapidly were implicated with the source and their actions prevented additional cases and deaths. Outbreak investigations also revealed unrecognized food sources and food safety gaps, resulted by regulatory and industry intervention [37].

Early diagnosis and antibiotic administration increase the probability of a favorable outcome. Also, due to the high mortality rate of listeriosis, it is necessary to reduce its incidence. For that purpose, it is urgent to identify the most efficient strategies based on risk assessment from the food production to consumption stage. According to the International Life Sciences Institute (ILSI), education is one of the main strategies to adopt reducing the incidence of listeriosis [45].

Another point to consider is that listeriosis is not a notifiable declaration disease in many countries. Thus, the actual incidence might be higher than the reported.

## 9. Conclusions

In summary, listeriosis is an important disease due to various factors. It is a common foodborne source and has the ability to survive and grow in hard conditions. We have to consider the long incubation period, the predilection for individuals who have an impairment of T-cell mediated immunity, and the atypical clinical manifestations that make it harder to make a diagnosis of* L. monocytogenes*. It is potentially fatal with a high mortality rate (20–30%) and it is higher in invasive listeriosis such as bacteraemia, despite a correct treatment. It is necessary to reduce its incidence. For that purpose, we have to identify the most efficient strategies based on risk assessment from the food production to consumption stage to reduce the incidence of listeriosis and make it a notifiable disease.

## Figures and Tables

**Table 1:** Mortality of* L*. *monocytogenes. *

Countries	Years	Death (%)
USA	2009–2011	17.6%
China	1964–2010	26%
Denmark	1994–2003	21%
Spain	2011	20–30%
Majorca	2002–2012	25%
Madrid	1986–2007	24.3%
Barcelona	2011	14%

**Table 2:** Incidence of listeriosis in the different studies.

Countries	Years	Incidence
USA	2004–2009	0.27
	2009–2011	0.29–1.3*
Taiwan	1996–2008	2.9
Denmark	1994–2003	0.46
	2011	0.88
Spain	1986–2007	0.46–1.03*
	2011	0.79

*Over 60 years old.
